# The 50 Most Cited Papers on Rugby since 2000 Reveal a Focus Primarily on Strength and Conditioning in Elite Male Players

**DOI:** 10.1155/2023/6991769

**Published:** 2023-12-19

**Authors:** Katherine J. Hunzinger, Eric Schussler

**Affiliations:** ^1^Department of Exercise Science, Thomas Jefferson University, Philadelphia, USA; ^2^School of Rehabilitation Sciences, Old Dominion University, Norfolk, USA

## Abstract

We sought to conduct a bibliometric analysis and review of the most cited publications relating to rugby since 2000 in order to identify topics of interest and those that warrant further investigations. Clarivate Web of Science database was used to perform a literature search using the search term “rugby.” The top 200 papers by citation count were extracted and reviewed for the inclusion criteria: all subjects were rugby players. The top 50 manuscripts were included for analysis of author, publication year, country of lead authors, institution, journal name and impact factor, topic, participant sex, and level of rugby. The total number of citations was 9,071 (average of 181.4 citations/article), with an average journal impact factor of 7.21; the top article was cited 407 times at the time of analysis. The most frequent publication was the Journal of Strength and Conditioning Research (26%), followed by the British Journal of Sports Medicine (20%) and the Journal of Sports Sciences (18%). Forty-eight (96%) of the manuscripts contained only male subjects, with 1 manuscript including females only and 1 manuscript containing mixed sexes. Thirty-three (66%) of the manuscripts focused on professional rugby players, with the next highest player group being mixed levels (10%). Twenty-eight (56%) concentrated on topics regarding strength and conditioning, 11 (22%) on injury, and 4 (8%) on physiology. Despite rugby being one of the most injurious sports and community players representing the largest component of the player pool, most of the top-cited rugby articles are cohort studies of professional male athletes focused on performance and strength and conditioning, noting the bias in research towards socially relevant topics that may not impact the majority of stakeholders and long-term health of rugby athletes. These findings highlight the need for further research among women and community athletes and on topics in injury prevention.

## 1. Introduction

Rugby is the most popular team collision sport, with 9.6 million players in over 123 countries, including 2.7 million female players [1]. Specifically, participation numbers tend to be highest in Europe and Africa, with the most prominent individual countries participating being England, Australia, South Africa, and other Commonwealth countries [1].

In recent years, the World Rugby Federation has created several initiatives to increase women's participation and equity in rugby, including a strategic initiative with the International Working Group on Women & Sport in 2022 [2]. Since 2014, the number of female players has increased by 57%, with a fourth of registered players being women and girls [3]. Despite these advances in participation, most of the literature has focused on male athletes despite being well established that male and female athletes have differing movement demands [4], response to injury [5], and general injury risk [6, 7]. These differences underscore the need to study both male and female athletes participating in rugby [8].

In tandem with the need to determine the presence of gender disparities in rugby research and the ever-growing popularity of rugby worldwide, a need exists to continue to research rugby through the lens of epidemiology, sports medicine, and injury prevention, as these data will produce insights for rugby stakeholders and sports medicine professionals to make informed decisions. In particular, rugby has the highest rate of concussion [9], paired with high incidence of musculoskeletal injuries [10], both of which are associated with physical and cognitive dysfunction in later life [11–13]. Therefore, a need exists to analyze the current state of the literature within rugby to determine trends in research topics and areas of focus to better understand any bias in research topics as well as any potential gaps for future research. By highlighting gaps in the literature, researchers will be better informed on the needs of rugby stakeholders as they prepare to secure funding and conduct research with the overall goal of improving the health and safety of rugby.

Network analysis using a keyword occurrence can offer a thorough understanding of the current research on rugby [14]. Using keyword occurrence, researchers can determine which topic areas have had the largest impact in their respective fields. This can be accomplished by determining the connections between common keywords and keyword combinations used in current research [15, 16]. Defining keyword occurrence volume and clustering can highlight important trends in the research such as heavily investigated and emerging topics [14]. Contrarily, these trends will help note gaps in the literature and identify needs for future research and funding opportunities.

Therefore, the purpose of this study was to determine the 50 most-cited articles about rugby since the year 2000. A secondary objective was to determine and/or visualize authors, countries, and keywords relating to the top 50 articles. Given that rugby originated in England and has the highest concussion rate of any sport [9], we hypothesized that the majority (i.e., greater than 50%) of the top-cited research would be conducted in Commonwealth countries and focus on sport-related concussions.

## 2. Methods

As a means of including all research pertaining to rugby, a literature search using only the search term “rugby” was conducted on the Clarivate Analytics Web of Science database on May 5, 2023. No restrictions on language, journal, country of origin, or article type were utilized. The only search restriction was the publication date on or after January 1, 2000.

**Figure 1:**
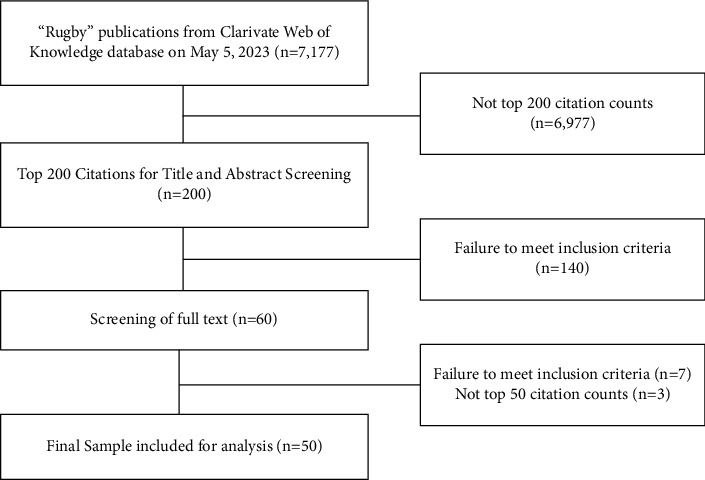
Flowchart of literature screening.

The search produced 7,177 articles which were then sorted from highest to lowest by number of citations. From this, the top 200 articles were pulled to be reviewed for their relevance to rugby for inclusion in the top 50 most cited articles list. All 200 articles were imported into Covidence software for both authors (KJH and ES) to independently screen for inclusion/exclusion based on title and abstract. Articles were included if the sample consisted entirely of rugby athletes at any competitive level. Articles were excluded if they were summary and/or position statements, editorials, systematic reviews, or meta-analyses. From this, 60 manuscripts remained, and both authors reviewed the full texts for consensus on meeting inclusion criteria. Seven manuscripts were excluded after a full-text review, leaving 53 manuscripts. From this, the top 50 cited out of the remaining 53 were accessed for inclusion and analysis (Figure 1).

The top 50 manuscripts were then reviewed to obtain variables of interest: first and/or senior author name, institutional affiliation (of first and/or senior author), journal name, number of article citations, keyword, participant sex, and level of competition. Additionally, the journal impact factor was obtained via Clarivate Journal Citation Reports.

Consistent with previous research [17, 18], citation density was calculated for each article as the number of total citations divided by the number of years since initial publication. Consistent with prior research [18] and for ease of organization, all data organization and summary statistics were computed using Microsoft Excel (Microsoft 365 MSO (Version 2304), Microsoft Corporation, Redmond, WA). Moreover, similar to previous research [17–19], due to open access and publicly available nature of these data, institutional review board approval was not required and thus not obtained for this study. Network analysis descriptions are provided below using established methods [19, 20].

### 2.1. Network Analysis

Keyword co-occurrence was utilized to identify keywords that appear in multiple articles and display their appearance volume. Author keywords and KeyWords Plus keywords provided by Web of Science were used in the analysis. KeyWords Plus keywords were included in the study since articles published in the British Journal of Sports Medicine did not include keywords. A minimum of 3 keyword appearances was selected for inclusion. A 3 keyword minimum was selected to remove keywords that did not connect to the keyword network and reduce the complexity of the visualization to allow it to be interpretable by selecting more influential keywords to display.

The record of articles and associated keywords was extracted from Web of Science with all listing details and imported into VOSviewer for bibliometric analyses (Version 1.6.19; VOSviewer, Leiden University, Leiden, Netherlands) [15]. Network analysis visualization was used for all maps whereby a circle visualizes items, the size of which is determined by its weight (i.e., the higher weight, the larger the circle). Circle colors are chosen by the cluster it belongs to, and lines between circles represent links. The closer the circles are to one another, the stronger they are related [20]. The dot size visualizes the number of connections in each image.

## 3. Results

The top 50 most cited articles about rugby research since January 1, 2000, and their respective citation counts and densities are presented in Table 1. The average number of citations was 181.4 ± 72.4 (median: 147), ranging from 122 to 407 total citations. The top citation density was found for Hulin et al. [26] at 39.0 citations/year since publication, with the lowest at 6.0 citations/year since publication for Baker [60]. The average citation density per article was 12.6 ± 6.1 citations/year since publication (Table 1).

Descriptively, the majority of the articles focused on professional athletes (66%), male athletes (96%), and topics about strength and conditioning/player performance (58%) followed by injury (22%). Female athletes were the focus of only 1 article, and mixed female and male athletes were the focus of 1 article. The included articles were published by 138 authors from 7 countries, with Australia contributing the most articles (articles = 25; 50%) and total number of citations (citations = 4,324; 47.7%) (Table 2).

In total, these manuscripts were written by 138 authors, with Gabbett having the most included articles (9) for a total of 1,623 citations, followed by Fuller CW, Kemp SPT, and Coutts A with 5 articles each with 1,264, 1,261, and 842 citations, respectively (Table 3).

The top 50 cited articles were published across 13 journals, with Impact Factors (IFs) ranging from 2.011 to 18.479 with an average IF of 7.206 ± 5.756 (median: 4.415). The Journal of Strength and Conditioning Research had the most included articles (26%) with 2,520 total citations (27.8%), followed by the British Journal of Sports Medicine (20%) with 1,977 total citations (21.8%) (Table 4).

**Figure 2:**
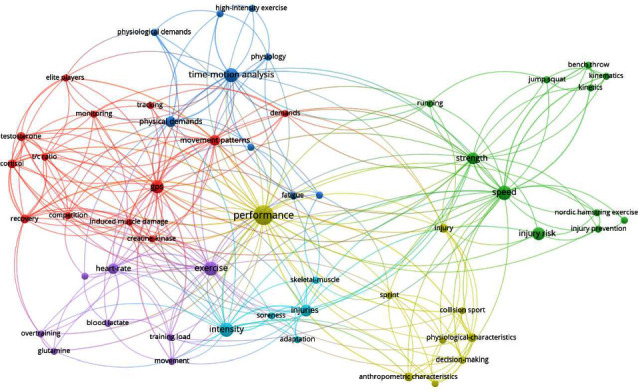
Keyword analysis. ^*∗*^Each keyword with 3 or more occurrences was included for analysis.

Keyword co-occurrence network mapping identified seven primary areas of research. The primary areas focused on performance, performance testing, outcomes, anthropometrics, time-motion analysis, physiologic outcomes of exercise, intensity, and injuries (Figure 2).

## 4. Discussion

This study aimed to conduct a bibliometric analysis of the most influential rugby papers since 2000. Our hypothesis was only partially supported, whereby most of the research was conducted in Commonwealth countries (90%); however, contrary to our hypothesis, only one article solely focused on concussion (2%), with 9 other articles including concussion as an outcome of interest, but not the sole outcome. Indeed, most research focused on strength and conditioning and athletic performance (58%). Moreover, secondary findings demonstrated that the majority of the top cited research focused on elite male athletes and were published in the early 2000s in journals such as the Journal of Strength and Conditioning Research and the British Journal of Sports Medicine. Overall, these findings highlight the bias towards male athletes in research, citing outdated research (e.g., citing papers referenced by other authors instead of finding more current research to cite), and the need for diversity in authorship and research topics given the current needs of rugby worldwide towards improving the health, safety, and long-term development of players.

The finding that most of the top 50 articles focused on strength and conditioning and athletic performance is of interest, given the relative popularity and monetary gain of professional rugby compared to other professional sports (e.g., National Football League and English Premier League Football). For reference, the 2021 total equity of World Rugby was £108 million ($138.5 million) [71], compared to the National Football League which has an estimated combined value of $142.87 billion [72]. Yet, this focus on performance may be an attempt by researchers and stakeholders (including study sponsors such as Rugby Football Union and World Rugby) to determine performance advantages for future sporting competitions. Interestingly, this study's total number of citations is less than in other sports, such as ice hockey [18] and American football [17]. This may result from the relative popularity and international reach of rugby compared to these other sports, with rugby only recently being readded to the Olympic sporting lineup in 2016. Moreover, American football and ice hockey have been highly cited likely due to the rise in sport-related concussion research and the ease of data collection in these cohorts due to the relatively high incidence of concussion and sample sizes [9, 73]. Unfortunately, it is not possible to truly determine why the top cited papers are on topics on strength and conditioning without speculating since there are many unknown factors that may lead to this publishing bias, including but not limited to a potential bias towards funding grants on topics in strength and conditioning resulting in more published data, greater acceptance of these topics by major journals, and/or greater reader interest in these topics resulting in higher citation counts. The current state of rugby research would benefit from published metrics from funding agencies on funding success rates by topic and highlighting the need for research on specific topics (i.e., player welfare and concussions).

In addition to the finding that the majority of top cited research was on strength and conditioning, a surprising finding was that minimal top research was focused on topics pertaining to sport-related concussion, given that rugby has one of the highest rates of concussion [9], and long-term player health. In recent years, data have shown that rugby players are at increased risk of neurodegenerative disease compared to the general population [74], with playing career length associated with risk of chronic traumatic encephalopathy [75]. Moreover, concussion history in rugby has been associated with worse patient-reported outcomes [76], lower-extremity injury [77, 78], worse outcomes of anxiety, depression, and sleep [79], and higher prevalence of irritability [79]. Given the current goal of World Rugby to advance the welfare and long-term health of athletes, there is a need to increase number of and improve the accessibility of studies pertaining to sport-related concussion in rugby. Importantly, it would benefit rugby stakeholders for researchers to publish their manuscripts in open access forums to improve the readability and translational effect of their research.

Herein, most of the research was published within Commonwealth countries which is expected given that rugby originated in England, and the top performers, participation rates, and competitions on the international stage are located within Commonwealth countries. Consistent with other bibliometric analyses on topics in sport [17, 18], the majority of the included studies were observational (44; 88%).

Professional male athletes were the most studied level of competition and sex among the top rugby-related publications since 2000. This is partially expected given that men represent the majority of the player pool and women are historically underrepresented in research on contact-sport athletes [80]. Unexpectedly, most of the research focused on professional athletes despite the average registered player being an amateur athlete [81]. Thus, these findings suggest a bias towards elite-level male players and indicate the need to expand research to other levels of players with a concerted effort towards female athletes. Moreover, the differences in research populations may result from research funding, with elite rugby either attracting more research, funding the research, and/or presenting a more accessible opportunity for study recruitment and participation.

Overall, 25 (50%) of the top cited manuscripts were published between 2000 and 2008, not including 2008. This is unexpected as rugby has grown exponentially in the last decade, with public awareness noting the long-term consequences of rugby participation [74, 76, 82]. Contrarily, this may be another incident result of publishing bias, whereby previous, and potentially dated, research was cited by known researchers and thus subsequently cited in modern research in lieu of more updated and current studies. Indeed, most of the top 50 ice hockey and American football publications in the last two decades have focused on sport-related concussions [17, 18]. Given that rugby has the highest rate of concussion [9], the lack of focus on concussion in the most cited rugby manuscripts in the last two decades is alarming.

Tim Gabbett from the Brisbane Broncos and the Tasmanian Institute of Sport and Colin Fuller from World Rugby were the top 2 authors in top rugby literature from the last two decades; these authors were included in 9 and 5 articles, respectively. It is unsurprising that these authors are highly cited given their respective decade long careers accompanied by successful funding, research collaborations, and partnerships with rugby teams (i.e., Brisbane Broncos and England Rugby Football Union). Both authors boast over 100 peer-reviewed publications with Dr. Tim Gabbett having over 36,000 lifetime citations. From an institutional level, the top institution of the lead author producing the included research was the University of Technology Sydney (5; 10%), followed by Rugby Football Union (3; 6%) and the University of Bath (3; 6%). Again, it is unsurprising that these institutions are involved with some of the top cited research given their locations in rugby-dominant countries and/or relationships with rugby teams and stakeholders (i.e., Rugby Football Union in England).

Regarding journal impact, the Journal of Strength and Conditioning Research (IF: 4.415) had the most publications, followed by the British Journal of Sports Medicine (IF: 18.479). The articles published in these two journals predominantly focused on strength and conditioning, the most highly cited topic, and rugby-related injuries. Given the highly cited nature of these publications and their relatively high IFs, it is recommended that researchers publish manuscripts pertaining to rugby strength and conditioning in the Journal of Strength and Conditioning Research and research pertaining to rugby-related injuries in the British Journal of Sports Medicine. Moreover, it is probable that these journals represent some of the top cited research in the field due to the fact that they have high impact factors, resulting in greater reach, increased likelihood of citation, and increased readership. Indeed, every journal included in this analysis has a scope exclusively focused on medical topics pertaining to sports medicine. Lastly, it is possible that rugby research tends to be published in journals based out of the United Kingdom like the British Journal of Sports Medicine. This is likely due in part to the fact that the United Kingdom has established rugby researchers, as noted by this study, a large rugby fan base [1] and existing relationships with teams to conduct research [83–85].

A surprising finding was that the majority of keyword co-occurrence surrounded “performance”. Indeed this hub term included subordinate nodes related to athletic performance, including injury risk, strength and speed, exercise, and performance analysis. Additionally, as noted above, most of this performance improvement-related research involved elite-level players, likely due to the level of resources available and financial benefit of elite team performance on the international level [3, 86]. Research surrounding rugby primarily improves player performance including the necessary components to keep players physically competing at their highest level. This outcome mirrors previous research, indicating that physical performance is a key research term across athletics [87]. Yet, this reference group needs to include more research on athletes' psychological well-being or game strategy. Given the negative long-term consequences associated with sport cessation and repetitive head impacts, future research should incorporate psychological and emotional well-being measures [12, 88, 89]. Moreover, there are negative financial consequences to injured players [90, 91], and given that rugby has some of the highest rates of concussion and musculoskeletal injury [9, 92], more research should be targeted towards topics on player well-being, injury prevention, and rehabilitation.

This study is limited by its methods, whereby ascertainment of the included manuscript using citation count may lead to misrepresenting the literature. For instance, the majority of the literature was before 2008, whereby given the relative age of the studies, it may have led to larger citation counts compared to newer, more recent studies. In addition, the citation count may result from funding disparities, positive outcome bias, and institutional and/or author prestige. We attempted to account for this bias by reporting citation density, highlighting differences in the top 50 rank location. Moreover, by limiting our study search to articles published in the 21^st^ century, we have missed more pivotal papers in the field that were published before the year 2000. However, we intentionally focused on more recent research to highlight more current trends in rugby research. Additionally, this study is limited by the use of a single database. Although this database has been commonly used in other bibliometric analyses [17, 18], other databases such as PubMed or Scopus may have produced different results. Collectively, these methods may have affected the overall sample of manuscripts included which in turn could have affected the breakdown of topics of research, authors, author countries, and journals where the research was published in.

## 5. Conclusions

This study determined that most of the top cited research on rugby published between 2000 and 2023 focused on professional male athletes and sport-related performance outcomes and originated from Commonwealth countries. This top 50 list will provide clinicians, researchers, and rugby stakeholders with a reference list of some of the most influential academic contributions to the research on rugby. Importantly, it highlights a clear gap in the literature and the need to fund, conduct, and publish research on amateur female rugby players in non-Commonwealth countries. Collectively, these findings highlight the bias in citation trends and topics among rugby research but also the need for further research among women and community athletes and on topics in injury prevention.

## Figures and Tables

**Table 1:** Top 50 most cited publications on rugby since 2000.

Rank	Title	Total citations	Year of publication	Citation density
1	Strength and power predictors of sports speed [21]	407	2005	22.6
2	Epidemiology of injuries in English professional rugby union: part 1 match injuries [22]	404	2005	22.4
3	Incidence, risk, and prevention of hamstring muscle injuries in professional rugby union [23]	379	2006	22.3
4	An evaluation of the physiological demands of elite rugby union using global positioning system tracking software [24]	317	2009	22.6
5	Neuromuscular, endocrine, and perceptual fatigue responses during different length between-match microcycles in professional rugby league players [25]	291	2010	22.4
6	The acute:chronic workload ratio predicts injury: high chronic workload may decrease injury risk in elite rugby league players [26]	273	2016	39.0
7	Physical demands of professional rugby league training and competition using microtechnology [27]	248	2012	22.5
8	Effects of resistance exercise combined with vascular occlusion on muscle function in athletes [28]	244	2002	11.6
9	Speed, change of direction speed, and reactive agility of rugby league players [29]	233	2008	15.5
10	Movement and physiological match demands of elite rugby league using portable global positioning systems [30]	221	2011	18.4
11	Effectiveness of post-match recovery strategies in rugby players [31]	214	2006	12.6
12	The physical demands of elite English rugby union [32]	211	2008	14.1
13	The effect of different warm-up stretch protocols on 20-meter sprint performance in trained rugby union players [33]	209	2004	11.0
14	Postactivation potentiation in professional rugby players: optimal recovery [34]	202	2007	12.6
15	Epidemiology of injuries in English professional rugby union: part 2 training injuries [35]	197	2005	10.9
16	Comparison of lower body strength, power, acceleration, speed, agility, and sprint momentum to describe and compare playing rank among professional rugby league players [36]	196	2008	13.1
17	Time-motion analysis of professional rugby union players during match-play [37]	190	2007	11.9
18	Testosterone, cortisol, and women's competition [38]	184	2002	8.8
19	Changes in selected biochemical, muscular strength, power, and endurance measures during deliberate overreaching and tapering in rugby league players [39]	165	2007	10.3
20	Physiological characteristics of junior and senior rugby league players [40]	162	2002	7.7
21	Time motion analysis of 2001 and 2002 super 12 rugby [41]	160	2005	8.9
22	A prospective study of injuries to elite Australian rugby union players [42]	159	2002	7.6
23	Contact events in rugby union and their propensity to cause injury [43]	155	2007	9.7
24	Influence of training and match intensity on injuries in rugby league [44]	153	2004	8.1
25	Sprint patterns in rugby union players during competition [45]	147	2006	8.6
26	Physical fitness qualities of professional rugby league football players: determination of positional differences [46]	147	2001	6.7
27	Comparison of upper-body strength and power between professional and college-aged rugby league players [47]	147	2001	6.7
28	Multiple effects of caffeine on simulated high-intensity team-sport performance [48]	146	2005	8.1
29	Relationship between training load and injury in professional rugby league players [49]	145	2011	12.1
30	Relationships between training load, injury, and fitness in sub-elite collision sport athletes [50]	143	2007	8.9
31	High school rugby players' understanding of concussion and return to play guidelines [51]	143	2006	8.4
32	The physical demands of super 14 rugby union [52]	141	2011	11.8
33	Evaluation of muscle damage after a rugby match with special reference to tackle plays [53]	141	2003	7.1
34	Tackle injuries in professional rugby union [54]	139	2008	9.3
35	Positional demands of international rugby union: evaluation of player actions and movements [55]	138	2013	13.8
36	The movement characteristics of English Premiership rugby union players [56]	137	2013	13.7
37	Monitoring for overreaching in rugby league players [57]	136	2007	8.5
38	Performance analysis of elite rugby league match play using global positioning systems [58]	132	2011	11.0
39	The prevalence, influential factors, and mechanisms of relative age effects in UK Rugby League [59]	132	2010	10.2
40	A series of studies on the training of high-intensity muscle power in rugby league football players [60]	132	2001	6.0
41	International Rugby Board Rugby World Cup 2007 injury surveillance study [61]	129	2008	8.6
42	The influence of in-season training loads on injury risk in professional rugby union [62]	126	2016	18.0
43	Biochemical and endocrine responses to impact and collision during elite rugby league match play [63]	126	2011	10.5
44	Inclusive masculinity theory and the gendered politics of men's rugby [64]	126	2010	9.7
45	A comparison of match demands between elite and semi-elite rugby league competition [65]	126	2009	9.0
46	Influence of fatigue on tackling technique in rugby league players [66]	125	2008	8.3
47	Eccentric knee flexor strength and risk of hamstring injuries in rugby union: a prospective study [67]	124	2015	15.5
48	Factors affecting perception of effort (session rating of perceived exertion) during rugby league training [68]	124	2013	12.4
49	Information-governing dynamics of attacker-defender interactions in youth rugby union [69]	123	2008	8.2
50	Surgical treatment of anterior instability in rugby union players: clinical and radiographic results of the Latarjet-Patte procedure with minimum 5-year follow-up [70]	122	2012	11.1

Citation counts as of May 5, 2023.

**Table 2:** Descriptive data of top cited rugby publications.

	Number of articles
Level of competition	
Professional	33
Mixed	5
Amateur	4
High school/youth	3
Semi-professional	3
College	1
Unknown	1
Sex of study participants	
Male	48
Female	1
Mixed	1
Topic of research	
Strength and conditioning/performance	29
Injury	11
Physiology	4
Sociology	2
Hamstring injury	2
Shoulder injury	1
Concussion	1
Country of origin	
Australia	25
United Kingdom and Ireland	15
New Zealand	5
Japan	2
United States of America	1
France	1
Portugal	1

**Table 3:** Descriptive data of the authors included in the most cited articles relating to rugby with two or more articles included.

Author name	Number of first or senior author publications	Number of middle author publications	Total number of articles included	Total number of citations	Average citations per publication
Gabbett	7	2	9	1,623	180.3
Fuller	5	0	5	1,264	252.8
Kemp	0	5	5	1,261	252.2
Coutts	3	2	5	842	168.4
Brooks	3	0	3	938	312.7
Reddin	0	3	3	980	326.7
Baker	3	0	3	475	158.3
Takarada	2	0	2	385	192.5
Duthie	2	0	2	307	153.5
Gill	1	1	2	352	176
McLellan	2	0	2	258	129
Sirotic	1	1	2	250	125

**Table 4:** Journals among the top 50 cited rugby articles with two or more articles.

Journal	Number of publications	2021 impact factor	Citations
Journal of Strength and Conditioning Research	13	4.415	2,520
British Journal of Sports Medicine	10	18.479	1,977
Journal of Sports Sciences	9	3.943	1,464
Journal of Science and Medicine in Sport	4	4.597	672
American Journal of Sports Medicine	3	7.01	642
International Journal of Sports Physiology and Performance	3	4.211	541
European Journal of Applied Physiology	2	3.346	380

## Data Availability

All data pertaining to this study are open access. A concise file of the included studies can be obtained by e-mailing the authors directly.
